# *Arecae pericarpium* extract induces porcine lower-esophageal-sphincter contraction via muscarinic receptors

**DOI:** 10.1186/s12906-021-03442-8

**Published:** 2021-11-04

**Authors:** Shu-Leei Tey, Chi-Ying Li, Li-Wei Lin, Li-Ching Chang, Yea-Ling Chen, Fang-Rong Chang, San-Nan Yang, Ching-Chung Tsai

**Affiliations:** 1 Department of Pediatrics, E-Da Hospital, No.1, Yi-Da Road, Yan-Chao District, Kaohsiung City, 82445 Taiwan, R.O.C.; 2 School of Medicine, I-Shou University, No. 8, Yi-Da Road, Yan-Chao District, Kaohsiung City, 82445 Taiwan, R.O.C.; 3 Graduate Institute of Natural Products, College of Pharmacy, Kaohsiung Medical University, No.100, Shih-Chuan 1st Road, Sanmin District, Kaohsiung City, 80708 Taiwan, R.O.C.; 4 School of Chinese Medicine for Post Baccalaureate, I-Shou University, No.8, Yi-Da Road, Yan-Chao District, Kaohsiung City, 82445 Taiwan, R.O.C.

**Keywords:** *Arecae pericarpium*, Lower esophageal sphincter, Motility, Muscarinic receptor

## Abstract

**Background:**

Gastroesophageal reflux disease (GERD) is associated with lower esophageal sphincter (LES) incompetence. In some patients, GERD is refractory to acid reduction therapy which is the main treatment for GERD. So far, medications that can increase LES tone are few. *Arecae pericarpium (A. pericarpium)* is a medication in Traditional Chinese Medicine known to promote intestinal motility.

**Methods:**

We investigated the effect of *A. pericarpium* extracts on porcine LES motility. In addition, we used tetrodotoxin (TTX) and atropine to study the underlying mechanism of *A. pericarpium* extracts-induced contractions of LES.

**Results:**

The results of this study showed that *A. pericarpium* extracts and their main active ingredient, arecoline, can induce the contractions of porcine LES sling and clasp muscles in a dose-response manner. TTX did not have an inhibitory effect on the contractions induced by *A. pericarpium* extracts and arecoline in LES. However, atropine significantly inhibited *A. pericarpium* extracts- and arecoline-induced contractions of LES.

**Conclusion:**

*A. pericarpium* extracts can induce the contractions of porcine LES in a dose dependent manner, possibly through muscarinic receptors, and hence, may be worth developing as an alternative therapy for GERD.

## Background

Gastroesophageal reflux disease (GERD) is a disorder involving inflammation of the lower esophagus which is mainly induced by food, bile, or acid regurgitation. Incompetence of the lower esophageal sphincter (LES), including decreased tone or repeated transient relaxation, is considered a major contributing factor to GERD [1, 2]. Up to 30% of patients show obstinate symptoms despite the use of proton pump inhibitors (PPIs), which reduce esophageal acid exposure [3]. This is partly due to the importance of LES incompetence in GERD. Presently, there are limited drug choices for the treatment of LES incompetence. Baclofen is one of the few drugs that can increase LES tone; however, due to multiple neurological side effects, the clinical application of baclofen in GERD is not feasible [4, 5].

*Arecae pericarpium (A. pericarpium)* is the dried pericarp of *Areca catechu* L*.* that is common in Southern China, India, Philippines, Taiwan, and Southeast Asian countries. The unripe fruit of *Areca catechu* L. is harvested from winter to spring, dried after cooking, and longitudinally split into two petals. Afterwards, the pericarp is peeled to obtain *A. pericarpium*, a medication in Traditional Chinese Medicine (TCM) embodied in Taiwan Herbal Pharmacopeia [6]. In accordance with the Compendium of Materia Medica, *A. pericarpium* has been used to treat constipation, abdominal distension, and edema in TCM. *A. pericarpium* has effect on gastric emptying and can promote the function of the small intestine in rats via muscarinic receptor [7, 8]. In addition, *A. pericarpium* can protect hepatic injury induced by alpha-naphthylisothiocyanate [9]. The use of *A. pericarpium* is considered safe and only very rare case experienced an allergic reaction after consumption. Furthermore, common dosage of *A. pericarpium* is 3–10 g [10].

To the best of our knowledge, there has been no study on the effect of *A. pericarpium* extracts on porcine LES motility to date. The aim of this study was to investigate the effect of *A. pericarpium* extracts on LES and the mechanism underlying A. *pericarpium* extracts-induced contractions of LES by using porcine LES.

## Methods

### Materials

This study was conducted in accordance with applicable laws and regulations of Taiwan, and E-Da hospital. All pigs weighed approximately 110 kg and were stunned with an electric shock device at 220 V for at least 3 s, followed by cutting off the main artery within 15 s of stunning, and exsanguination until death. All pigs were slaughtered in a regulated slaughterhouse supervised by the Council of Agriculture, Executive Yuan, R.O.C. (Taiwan). The stomachs and lower esophagi of pigs were purchased from this slaughterhouse in Kaohsiung City. The subjects were exempted from the review of Institutional Animal Care and Use Committee of E-DA Hospital because the esophagi and stomachs of pigs are considered pork variety meats and not live animal parts. After proximal stomachs and distal esophagi were obtained, they were delivered in an ice-cold oxygenated Kreb-Henseleit solution to the laboratory in 30 min. The Kreb-Henseleit buffer solution was composed of the following: 25 mM NaHCO_3_, 1.2 mM NaH_2_PO_4_, 4.7 mM KCl, 118 mM NaCl, 1.8 mM CaCl_2_, and 14 mM glucose, and was kept under constant pH of 7.4. The tetrodotoxin (TTX) was purchased from Tocris Cookson Inc. (Avonmouth, Bristol, UK), and arecoline hydrobromide and atropine were manufactured by Sigma-Aldrich (St. Louis, MO, USA).

### Preparation of *A. pericarpium* extracts

*A. pericarpium* was purchased from Da-Tian Chinese Medicine pharmacy (Kaohsiung, Taiwan, ROC) and authenticated by Dr. Li-Wei Lin (The School of Chinese Medicine for Post-Baccalaureate, I-Shou University) according to Taiwan Herbal Pharmacopeia [6]. A herbarium sample (code number, ISU-MCMM-199) was preserved in the School of Chinese Medicine for Post-Baccalaureate, I-Shou University (Kaohsiung, Taiwan, ROC) for future reference. Dried *A. pericarpium* (30 g) was chopped and soaked in 300 ml of 95% ethanol for 24 h and the mixture was filtered with gauze. The resulting filtrate is referred to as extract 1 of *A. pericarpium*. Residual *A. pericarpium* was soaked and filtered repeatedly in the same way as described above, to produce extracts 2 and 3, respectively. Additionally, 1 g of *A. pericarpium* was powdered, soaked in 20 mL of 95% ethanol, vortexed using ultrasonic power 200 W at 30 °C for 1 h, and the resulting solution filtered to produce extract 4. These extracts were concentrated under vacuum with a rotary evaporator to produce around 30 ml of dense plasters, and the plasters were then freeze-dried [11, 12].

### High performance liquid chromatography (HPLC) analysis of arecoline in *A. pericarpium* extracts

The chromatographic system consisted of a Shimadzu SIL-10 AD VP auto injector, a Shimadzu LC-20 AD prominence liquid chromatography and a Shimadzu SPD-M10A VP diode array detector (Shimadzu, Kyoto, Japan). A Cosmosil 5C18-PAQ 4.6 mm × 250 mm column (Nacalai tesque, CA, USA) was used for the separation and the temperature was controlled at 30 °C. The mobile phase consisted of 0.5% aqueous phosphoric acid and 99.5% acetonitrile at volumetric ratios. The detection was monitored at 215 nm. The mobile phase was delivered at a rate of 1.0 mL/min and the volume of injection loop was 10 μL. According to Taiwan Herbal Pharmacopeia, arecoline is the active ingredient of *Areca catechu* L. [6]. Arecoline (1 mg/mL) was diluted to 0.4, 0.2, 0.1, 0.05, 0.025, and 0.01X and analyzed using HPLC. We also dissolved 1 mg powder of extracts of *A. pericarpium* in 1 ml of 10% ethanol for HPLC analysis. The extraction yield of arecoline from *A. pericarpium* was calculated as

$$\mathrm{Extraction}\ \mathrm{yield}\ \left(w/w\right)=\frac{\mathrm{Mass}\ \mathrm{of}\ \mathrm{arecoline}\ \left(\mathrm{in}\ \mathrm{extracted}\ \mathrm{solution}\right)}{\mathrm{Mass}\ \mathrm{of}\ \mathrm{material}\ A. pericarpium}\times 100\%$$

### Measurement of *A. pericarpium* extracts-induced contractions of porcine LES

In contrast to the circular LES of rats, pigs share similar anatomy of LES with humans as they are both composed of sling and clasp muscles [13]. For this reason, swine LES is commonly used for the study of human gastroesophageal reflux and esophageal motility disorder [14–17]. According to previously reported method, one end of the muscle strips was attached to an isometric transducer with surgical wire (FORT10g; Grass Technologies, RI, USA). The transducer was connected to an amplifier (Gould Instrument Systems, OH, USA), the signal obtained was then recorded by a computer recording system (BIOPAC Systems, CA, USA) [16, 17]. The muscle’s basal tone was set to 1.0 g for this study. After a 30-min equilibration period, 1 × 10^− 6^ M carbachol was added to the organ bath to induce contraction of the muscle strip, and the carbachol was washed away. One mg powders from extracts 1 and 2 respectively were used and dissolved in 1 ml 10% ethanol in order to check the effect of *A. pericarpium* extracts on LES. One hundred μL (= 100 ng/L) of extract 1 of *A. pericarpium* was added after another 30-min equilibration period, and 200 μL (= 200 ng/L) of extract 1 of *A. pericarpium* was added (cumulative dose = 300 ng/L) when the contraction reached a peak equilibration induced by 100 μL of extract 1 of *A. pericarpium*. In the same way, the extract 2 of *A. pericarpium* was also used to check its effect on LES.

### Measurement of arecoline-induced contractions of porcine LES

In order to check the effect of arecoline on LES, 300 nM arecoline was added after a 30-min equilibration period, and 1 μM arecoline was added when the contraction reaches a peak equilibration induced by 300 nM arecoline.

### Effect of TTX on *A. pericarpium* extracts- and arecoline-induced contractions of porcine LES

To investigate the mechanism of *A. pericarpium* extracts- and arecoline-induced contractions of LES, the isolated LES was pretreated with TTX (10^− 6^ M) into the organ bath. After 15 min, *A. pericarpium* extract (100 ng/L and 200 ng/L) or arecoline (300 nM and 1 μM) was added sequentially to the organ bath as earlier described [16, 18].

### Effect of atropine on *A. pericarpium* extracts- and arecoline-induced contractions of porcine LES

To investigate the mechanism of *A. pericarpium* extracts- and arecoline-induced contractions of LES, the isolated LES was also pre-treated with atropine (10^− 6^ M), for 6 min followed by the addition of *A. pericarpium* extract (100 ng/L and 200 ng/L) or arecoline (300 nM and 1 μM) sequentially to the organ bath as earlier described [16, 18].

### Data analysis

The data are expressed as the means ± SEM. Statistical analysis of the results was performed by using Mann-Whitney U test. The minimum sample size was 4. In all cases, differences were considered significant at *p* < 0.05. All analyses were performed using the SPSS statistical software version 24 (IBM Corp., NY, USA).

## Results

### HPLC analysis of arecoline in *A. pericarpium* extracts

**Fig. 1:**
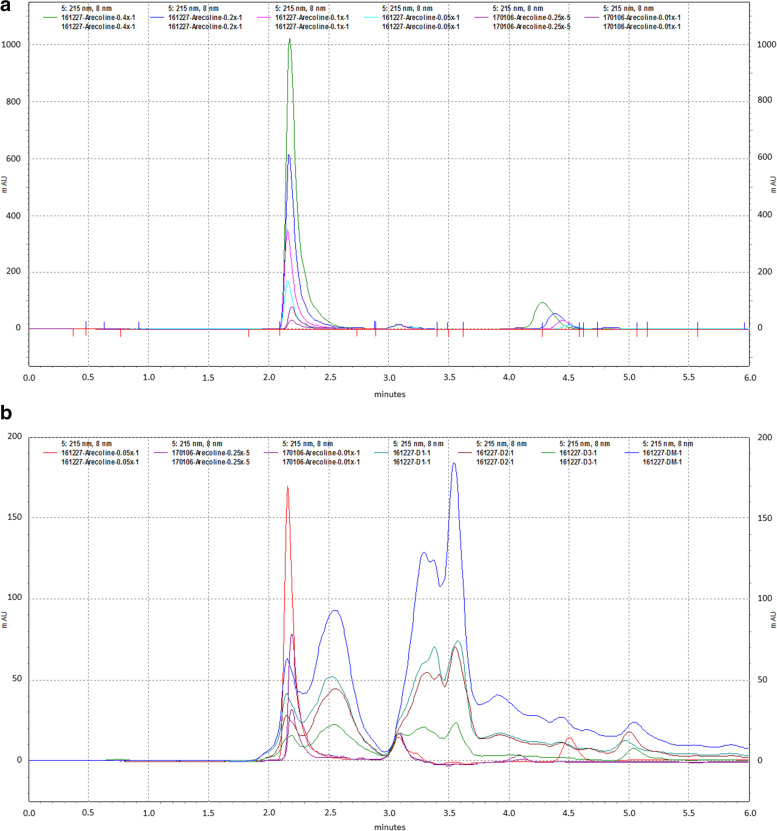
HPLC of arecoline and *Arecae pericarpium* (*A. pericarpium*) extracts. The chromatograms reveal (**A**) the repeatability and reproducibility of the arecoline standard, and (**B**) arecoline in separations of *A. pericarpium* extracts 1–4 (D1, D2, D3, and DM represent extracts 1, 2, 3, and 4, respectively)

The chromatogram of the standard compound arecoline and that of the extracts of *A. pericarpium* is shown in Fig. 1A and B, respectively*.* The sample calibration curve for arecoline was linear (r^2^ = 0.9982) within the range 0–50 mg/mL. Intra- and inter-day coefficients of variation of the assays were less than 5% (*n* = 5). The content of arecoline was detected by HPLC and quantified as 1.3, 0.6, 0.3 and 2.0 μg/mL in extracts 1, 2, 3, and 4 of *A. pericarpium,* respectively. Furthermore, because 0.47, 0.57, 0.81 and 0.16 g powders were obtained from extracts 1, 2, 3, and 4, respectively, the extraction yield of arecoline from *A. pericarpium* was 0.02, 0.011, 0.011, and 0.32% in extracts 1, 2, 3, and 4, respectively.

### Measurement of *A. pericarpium* extracts-induced contractions of porcine LES

**Fig. 2:**
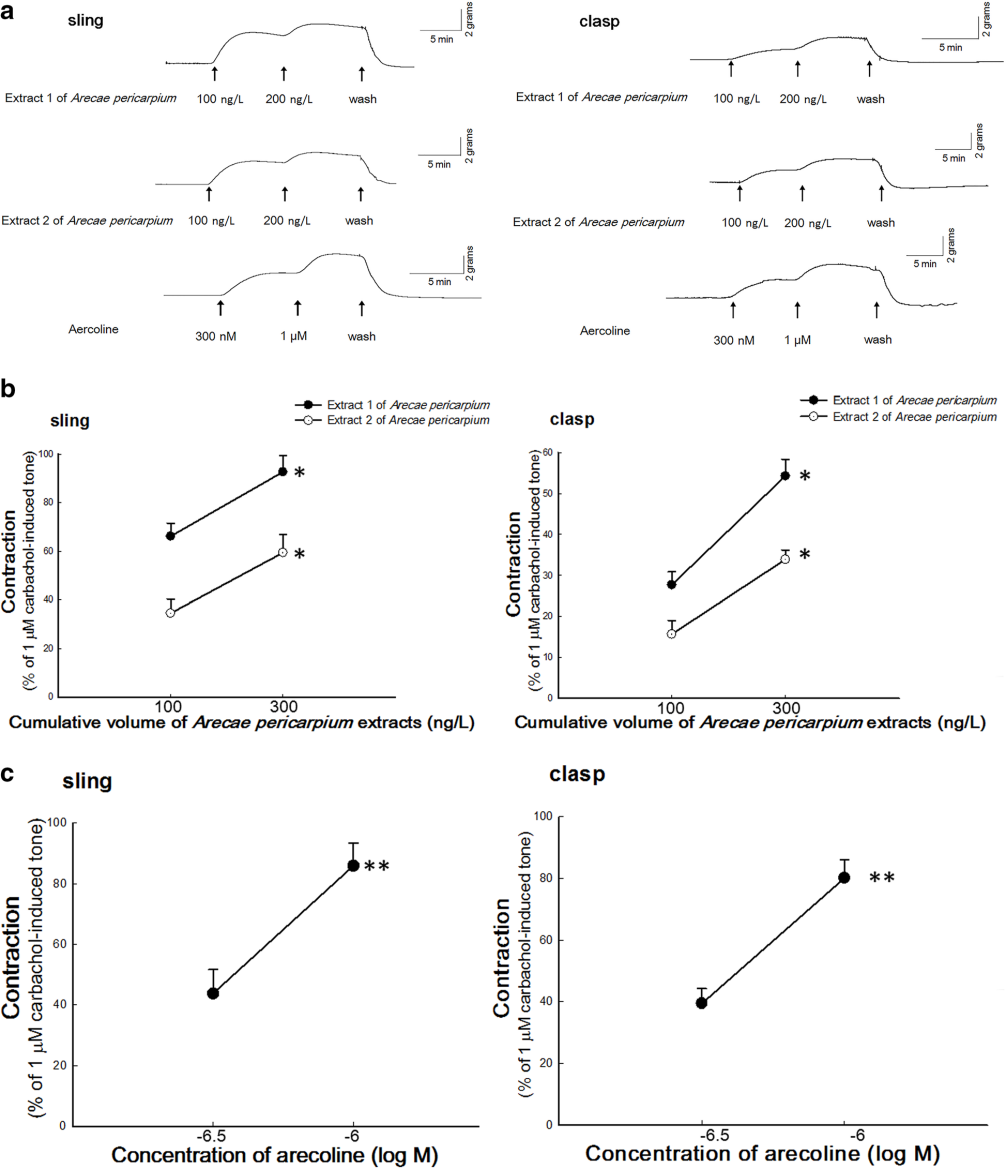
The *Arecae pericarpium* (*A. pericarpium*) extracts- and arecoline-induced contractions of porcine lower esophageal sphincters (LES). **A** Typical tracing of the contractions of porcine LES sling and clasp muscles in response to cumulative addition of *A. pericarpium* extracts and arecoline. Arrows indicate the addition of *A. pericarpium* extracts to LES as cumulative dosage. Dose-response curves of (**B**) extracts 1 and 2 of *A. pericarpium*-induced (*n* = 4) or (**C**) arecoline-induced (*n* ≥ 4) contractions of porcine sling and clasp muscles. *,** represent significant differences (*p* < 0.05) from the response caused by 100 ng/L corresponding *A. pericarpium* extract or 300 nM arecoline, respectively

Figure 2A shows typical tracings of *A. pericarpium* extracts-induced tonic contractions of sling and clasp muscles. Contractile responses were produced in sling and clasp muscles by the application of increasing dosages of *A. pericarpium* extracts. As shown in Fig. 2B, cumulative doses of 100 and 300 ng/L of extract 1 of *A. pericarpium* caused significant contractile responses of 66.19 ± 5.44% and 92.71 ± 6.61% while extract 2 caused 34.54 ± 5.79% and 59.43 ± 7.43% responses to 1 μM carbachol-induced contractions in sling muscles, respectively. There was a significant difference between the two contractions induced by different volumes of either extract 1 or 2 (*p* = 0.02 and 0.04 respectively, both *n* = 4). In addition, cumulative doses of 100 and 300 ng/L of *A. pericarpium* extract 1 caused significant contractions of 27.72 ± 3.18% and 54.30 ± 4.09% while extract 2 caused 15.69 ± 3.36% and 33.90 ± 2.32% responses to 1 μM carbachol-induced contractions in clasp muscles, respectively. Cumulative dose of 300 ng/L of either extract 1 or 2 induced an increased significantly contractions than 100 ng/L respectively (*p* = 0.002 and 0.005 respectively, both *n* = 4).

### Measurement of arecoline-induced contractions of porcine LES

Figure 2A shows typical tracings of arecoline-induced tonic contractions of sling and clasp muscles. As shown in Fig. 2C, the contractile responses of LES with cumulative doses of 300 nM and 1 μM arecoline were 43.82 ± 7.91% and 85.94 ± 7.58% while 39.41 ± 4.81% and 80.15 ± 5.92% contractions was induced by 1 μM carbachol in the sling and clasp muscles, respectively. There was a significant difference between the two contractions induced by 300 nM and 1 μM arecoline in LES sling and clasp muscles (*p* = 0.002 and 0.0002, *n* = 4 and 7 respectively).

### Effect of TTX on *A. pericarpium* extracts- and arecoline-induced contractions of porcine LES

**Fig. 3:**
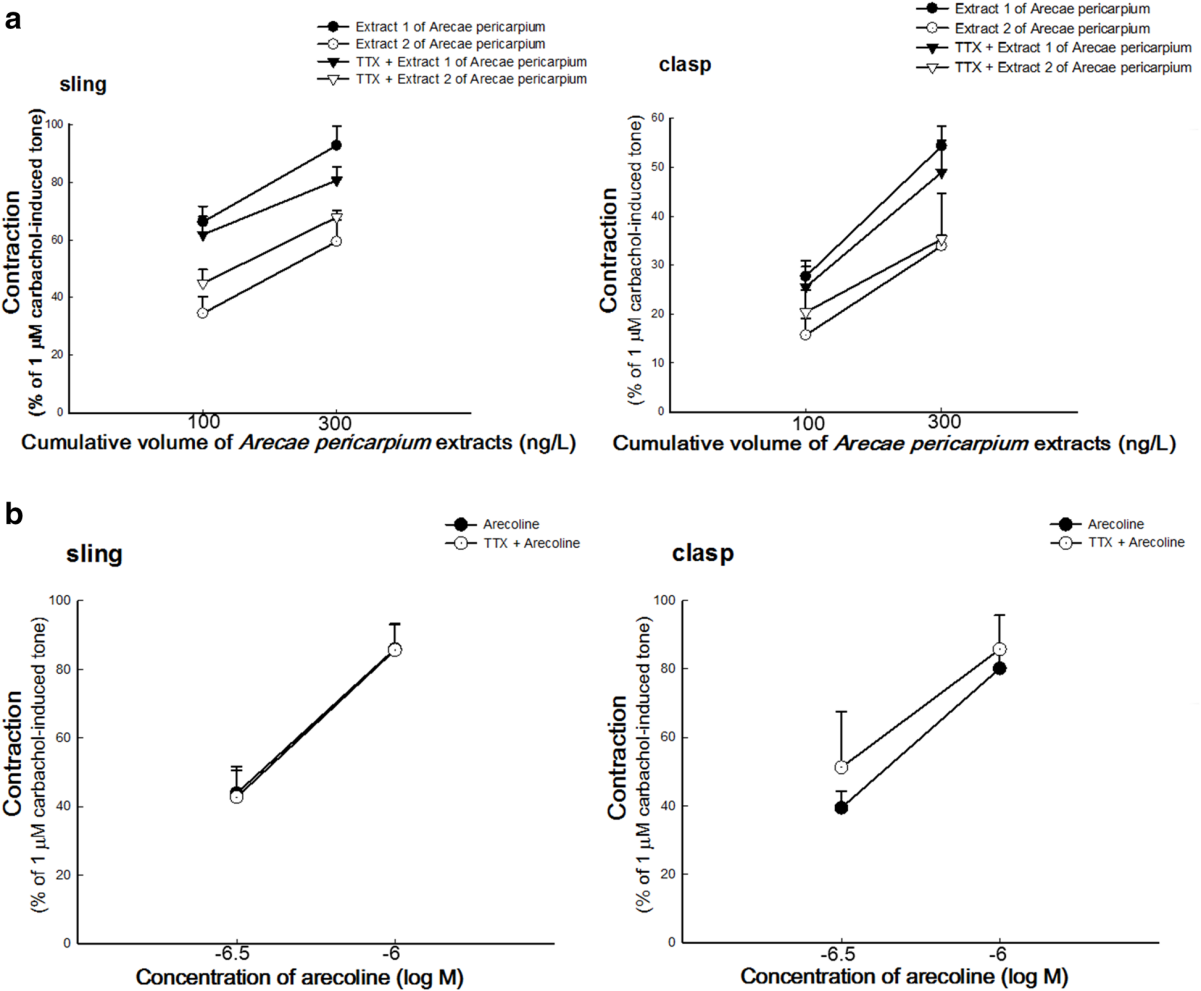
Effects of tetrodotoxin (TTX) on the contractions of porcine lower esophageal sphincters (LES) induced by *Arecae pericarpium* (*A. pericarpium*) extracts and arecoline. A concentration of 10^− 6^ M TTX had no significant inhibitory effect on the contractions of sling and clasp muscles induced by (**A**) extracts 1 and 2 of *A. pericarpium* (n = 4) or (**B**) arecoline (n ≥ 4)

As shown in Fig. 3A, the dose-response contractions of *A. pericarpium* extracts were almost unaffected by pretreatment with TTX (*p* > 0.05, compared to the corresponding *A. pericarpium* extracts alone, n = 4). In addition, the effect of TTX on arecoline-induced contractions of LES sling and clasp muscles was not significant, compared to arecoline alone in Fig. 3B (*p* > 0.05, *n* ≥ 4).

### Effect of atropine on *A. pericarpium* extracts- and arecoline-induced contractions of porcine LES

**Fig. 4:**
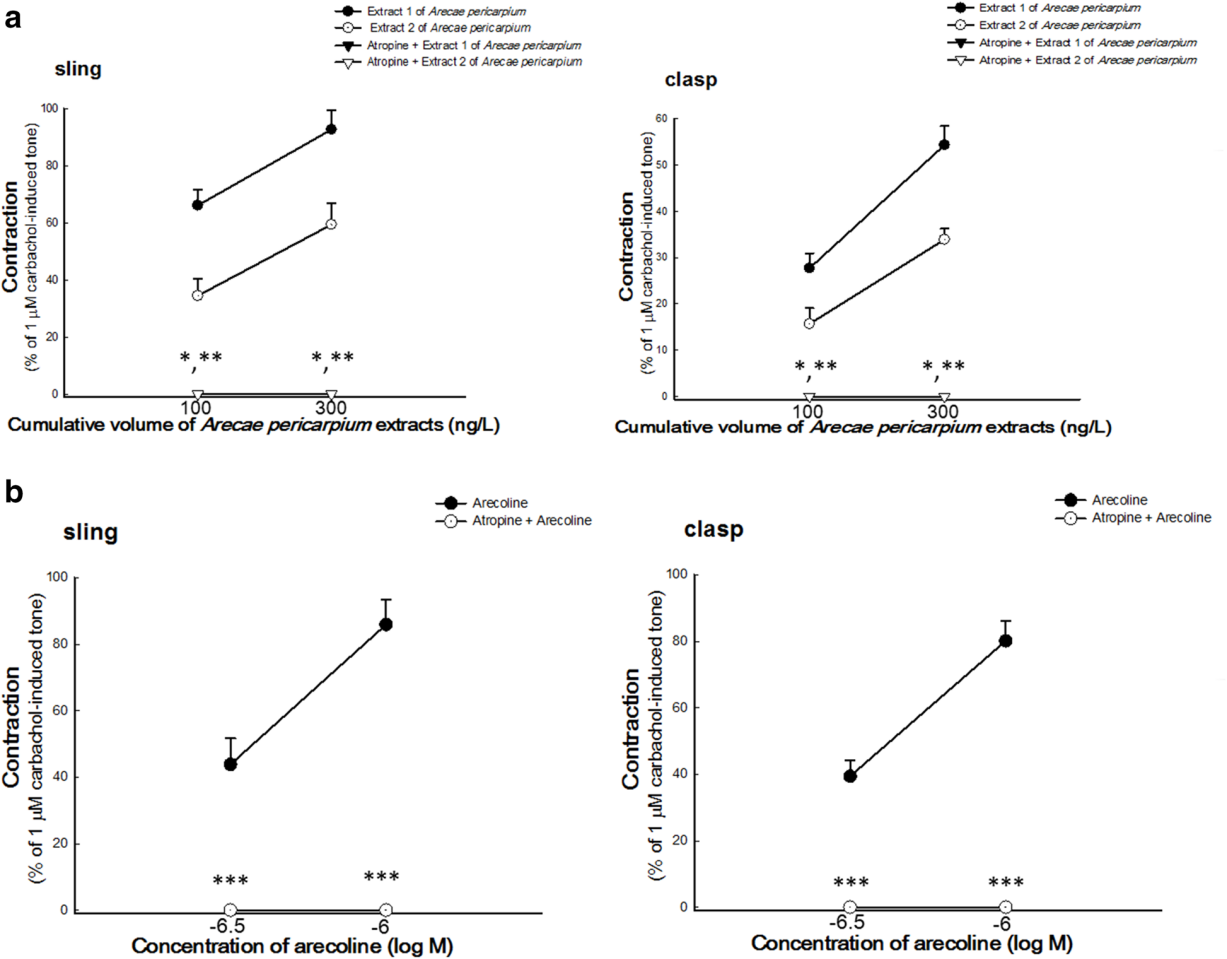
Effects of atropine on the contractions of porcine lower esophageal sphincters (LES) induced by *Arecae pericarpium* (*A. pericarpium*) extracts and arecoline. Atropine significantly inhibited (**A**) extracts 1 and 2 of *A. pericarpium*-induced (n = 4) and (**B**) arecoline-induced (n ≥ 4) contractions of porcine LES sling and clasp muscles. *, **, *** represent significant differences (*p* < 0.05) from the response caused by the corresponding cumulative volume of extract 1, 2, or arecoline only, respectively

As shown in Fig. 4A, pretreatment with atropine had a significant inhibitory effect on *A. pericarpium* extracts-induced contractions of LES (*p* = 0.001 (100 ng/L) and 0.0007 (300 ng/L) in sling muscles and 0.009 (100 ng/L) and 0.004 (300 ng/L) in clasp muscles, compared to *A. pericarpium* extracts alone, all *n* = 4). In addition, a significant inhibitory effect of atropine on arecoline-induced contractions of LES sling and clasp muscles was also observed, compared to arecoline alone as shown in Fig. 4B (*p* = 0.005 (300 nM) and 0.0003 (1 μM) in sling muscles and 0.0001 (300 nM) and 0.00001 (1 μM) in clasp muscles, n = 4 and 7 in sling and clasp muscles respectively).

## Discussion

These results reveal that *A. pericarpium* extracts and their main active ingredient, arecoline, caused the contractions of porcine LES sling and clasp muscles in a dose-response manner. In addition, LES contraction curves induced by *A. pericarpium* extracts and arecoline returned to the prestimulation levels after the removal of *A. pericarpium* extracts or arecoline, suggesting that these contractile effects probably did not occur via toxic effects.

Excitatory postganglionic myenteric neurons in the LES may release acetylcholine and its derivatives, which activate muscarinic receptors, thereby causing an increase in cytosolic [Ca^2+^] [19]. In addition, the activation of Ca^2+^ channels facilitates extracellular Ca^2+^ influx into cells, thereby increasing cytosolic [Ca^2+^]. Cytosolic Ca^2+^ binds to calmodulin, resulting in the activation of myosin light chain kinase (MLCK). MLCK phosphorylates the 20 kDa light chain of myosin, and myosin conjugates with actin to initiate cross-bridge cycling which induces smooth muscle contraction [20, 21].

TTX acts as a sodium channel blocker which blocks voltage-dependent sodium channels in motor neurons. However, the contractions of LES sling and clasp muscles induced by *A. pericarpium* extracts and arecoline were not blocked by TTX in this study. This indicates that *A. pericarpium* extracts- and arecoline-induced contractions of LES were not induced by the effect of these substances on nerve fibers.

The non-selective muscarinic receptor antagonist atropine can block the muscarinic receptors (M_1–5_). Atropine inhibited *A. pericarpium* extracts- and arecoline-induced contractions of LES sling and clasp muscles. These results indicate that *A. pericarpium* extracts- and arecoline-induced contractions were possibly mediated by muscarinic receptors.

Extracts from several medicinal plants, such as *Ceratonia silique*, *Myrtus communis, Salvia miltiorrhiza,* and *Cydonia oblonga*, have been reported to be useful for the management of GERD, in both animal and human studies. In these animal studies, the mechanisms underlying the beneficial effects of the extracts were considered to be related to anti-oxidation, anti-inflammation, improvement of gastric mucus and barrier function, or a reduction in gastric acid [22]. However, inducing LES contraction to avoid reflux is more helpful for patients with PPI-refractory GERD than other treatment effects. To date, only *Salvia miltiorrhiza* has been reported to induce tonic contraction of the LES in Sprague-Dawley rats [18]. However, the effect of *A. pericarpium* extract on porcine LES is more dominant than that of *Salvia miltiorrhiza* extract in rats because of the similarities between the porcine and the human LES.

Previous study demonstrated that arecoline can excite the colonic smooth muscle motility in rabbits via muscarinic receptor [23]. In addition, *A. pericarpium* can promote gastrointestinal motility in rats via muscarinic receptor [7, 8]. However, the LES is a specialized smooth muscle that is different from the muscularis propria of the gastrointestinal tract, because LES always remains contracted and temporarily opens only during swallowing. A key novel finding of this study is that *A. pericarpium* extracts and arecoline can induce contractions in this specialized smooth muscle.

Arecoline is one of several active ingredients of *Areca catechu* L. and can cause side effects such as the promotion of oral submucosal fibrosis (OSF). However, the development of OSF takes time, and it is observed that 3.5 and 6.5 years of chewing areca nut was necessary to develop OSF in younger and older cohorts, respectively [24]. Furthermore, the half-maximal inhibitory concentration (IC_50_) of arecoline was approximately 210 μM in HaCaT keratinocytes and buccal mucosal fibroblasts (BMFs) [25, 26]. The concentrations of arecoline used in studies on OSF were reported to be around 160 μM in HaCaT cells and 0 to 672 μM in BMFs [27, 28]. However, in this study, the working concentration of arecoline applied to LES tissues (300 nM to 1 μM) was much lower than previously reported IC_50_ concentrations or those used for studying OSF. Moreover, arecoline has different effects depending on the concentration, such as increasing cell proliferation rate but inducing cell cycle arrest, apoptosis, and DNA damage at lower and higher concentrations, respectively, in oral squamous cell carcinoma cells [29]. Previous studies have focused on the relationship between the dosage and toxicity of arecoline, but it is important to determine the difference between pharmacological dosages with an appropriate effect and toxic doses of arecoline [30].

In addition, epigallocatechin-3-gallate (extracted from green tea), *Ganoderma microsporum* immunomodulatory protein (extracted from *Ganoderma microsporum*), and hinokitiol (derived from *Chamacyparis taiwanensis*) can suppress arecoline-induced OSF [31–34]. These findings provide an opportunity for further research to develop a multicomponent herbal preparation that not only mitigates against unwanted side effects of arecoline but which can also be used for the management of GERD.

## Conclusions

*A. pericarpium* extracts can induce the contractions of porcine LES in a dose dependent manner, and the contractions are possibly related to muscarinic receptors. These results suggest that *A. pericarpium* may be exploited as a potential alternative therapy for GERD.

## Data Availability

All relevant data are included within the manuscript and are available from the corresponding author on reasonable request.

## Abbreviations

GERD: Gastroesophageal reflux disease
PPI: Proton pump inhibitor
LES: Lower esophageal sphincters; *A. pericarpium*: *Arecae pericarpium*
TCM: Traditional Chinese Medicine
HPLC: High performance liquid chromatography
MLCK: Myosin light chain kinase
TTX: Tetrodotoxin
IC_50_: Half-maximal inhibitory concentration
